# The Role of Gap Junctions in MSC-EA.hy926 (An Endothelial Cell Model) Crosstalk Under Hypoxic Stress: Regulation of the Angiogenic Response

**DOI:** 10.3390/ijms262211239

**Published:** 2025-11-20

**Authors:** Mariia Ezdakova, Diana Matveeva, Margarita Lobanova

**Affiliations:** Institute of Biomedical Problems, Russian Academy of Sciences, Moscow 123007, Russia; matveeva.dajana@yandex.ru (D.M.); pogodina_m@mail.ru (M.L.)

**Keywords:** angiogenesis, multipotent mesenchymal stromal cells (MSCs), EA.hy926 (an endothelial cell model), gap junctions (GJs), intercellular communication, hypoxia, tissue homeostasis

## Abstract

Effective communication between multipotent mesenchymal stromal cells (MSCs) and endothelial cells (ECs) plays a critical role in the regulation of angiogenesis, especially under conditions of hypoxia. In addition to paracrine stimulation, direct intercellular contacts play an important role in the angiogenic interaction between MSCs and ECs, making them an important target for modulating vascular network restoration under ischemic conditions. The aim of this study was to determine the contribution of gap junctions (GJs) to the angiogenic response of MSCs and the EA.hy926 cell line (an Endothelial Cell Model) under acute hypoxic stress. In a cell co-culture model at 0.1% O_2_ using a specific GJ inhibitor (carbenoxolone), molecular, cellular, and functional tests were performed: assessment of viability, proliferation, migration, secretion of angiogenic mediators, and expression of crucial genes. GJ blockade was accompanied by decreases in the proliferation and migration activity and angiogenic potential of the conditioned medium in in vitro and in ovo tests. These data highlight the importance of the GJ in coordinating the angiogenic response in conditions of acute hypoxia and can be used to develop protocols for regenerative medicine.

## 1. Introduction

The functioning of tissues and organs, both under conditions of physiological homeostasis and when damaged, is largely determined by the coordinated interaction of cells with their surrounding microenvironment. Of particular interest in this context is the formation of new blood vessels, which requires the coordinated activity of vascular wall cells and elements of the perivascular niche [1]. Co-cultivation of multipotent mesenchymal stromal cells (MSCs) and endothelial cells (ECs) is used as a promising model for studying the mechanisms of angiogenesis, since the interaction between these cells ensures the formation and maintenance of the vascular network in vivo [2,3,4].

Experimental data indicate that the interaction between MSCs and ECs in vitro contributes to increased angiogenic activity, including increased proliferation, cell migration, and secretion of angiogenic factors [5]. These effects are provided by both paracrine regulation and the formation of specialized structures of direct intercellular communication [6,7,8,9]. In particular, the functional significance of gap junctions (GJs) for the realization of a coordinated vascular response, including control of cell migration, proliferation, and differentiation, has been noted [8,10,11]. Inhibition of GJs leads to a decrease in the angiogenic potential of MSC and EC associates—the biological activity of their conditioned medium decreases and angiogenesis in ovo is suppressed [9] GJs synchronize cellular activity [8,12,13,14] through direct exchange between the cytosols of neighboring cells of ionic and molecular signals—calcium ions, metabolites, amino acids, secondary messengers, microRNAs, etc. [15,16,17]. This makes them an important target for studying cellular responses to environmental factors.

Among factors affecting the effectiveness of intercellular communication, oxygen levels are particularly important. Local hypoxia is a frequent companion of pathological conditions such as ischemia, inflammation, and tissue trauma, and serves as a trigger for angiogenesis, which is necessary to restore the metabolic needs of damaged tissue [18,19,20,21]. Studies of acute hypoxia on the behavior of MSCs and ECs in angiogenic processes reveal several trends. On the one side, hypoxic stress can disrupt the proliferation and viability of these cells [22,23,24]. On the other side, hypoxia boosts their trophic activity by stimulating the secretion of angiogenic factors such as VEGF-A, FGF-2, and ANGPT1 [25]. However, to date, there are no systematic studies evaluating the contribution of the GJs to the regulation of MSC-EC interaction under conditions of acute hypoxia. Understanding the role of heterocellular communication via GJs under low O_2_ conditions may be fundamentally important for developing new therapies for ischemic and inflammatory diseases.

In this study, we focused on the role of functional gap junctions (GJs) between MSCs and endothelial-like cells under conditions of acute hypoxic stress (<1% O_2_), which models the acute phase of ischemic injury characterized by a sharp decrease in oxygen levels and the activation of rapid adaptive mechanisms of intercellular communication. As an endothelial model, we used the EA.hy926 cell line — a hybrid of human umbilical vein endothelial cells (HUVEC) and the lung adenocarcinoma cell line A549 — which retains many phenotypic and functional characteristics of endothelial cells and is widely used as an endothelial-like model in vascular research. We hypothesize that GJs modulate the angiogenic response, by ensuring coordinated cell adaptation and maintaining tissue homeostasis during oxygen deficiency. Studying the contribution of GJs under hypoxic conditions may be an important step toward a deeper understanding of the mechanisms of angiogenesis in ischemic lesions and the discovery of new therapeutic strategies aimed at modulating intercellular communication.

## 2. Results

### 2.1. Cellular Response to Hypoxia

Cells respond to changes in oxygen levels by activating genetic programs aimed at adapting to hypoxic conditions. These programs include the expression of genes involved in angiogenesis, glycolysis, metabolic restructuring, and the regulation of cell proliferation, survival, and apoptosis. The central molecular mediator of the cellular response to hypoxia is hypoxia-inducible factor-1α (HIF-1α), which plays a key role in maintaining oxygen homeostasis [26].

**Figure 1:**
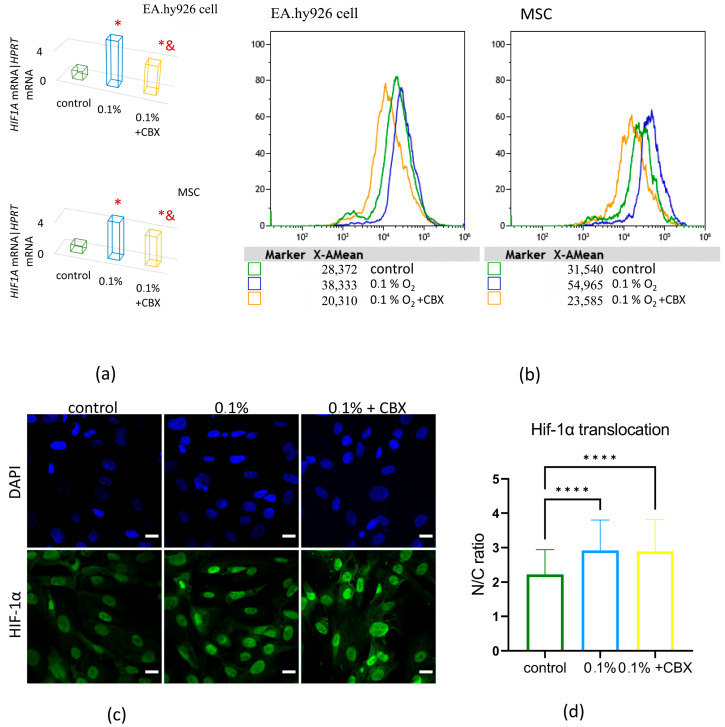
HIF-1α expression increases in response to hypoxic exposure. (**a**) Relative mRNA expression of *HIF1A* measured by qRT-PCR; Data are presented as mean ± SD, *n* = 4, * *p* ≤ 0.05 vs. control (20% O_2_); & *p* ≤ 0.05 vs. 0.1% O_2_; (**b**) Representative histograms, flow cytometry. Distribution of cells based on mean fluorescence intensity (MFI). HIF-1α protein level in hypoxia-treated cells stained with anti-HIF-1α antibodies; (**c**) Immunofluorescent staining of HIF-1α in co-culture cells analyzed by confocal microscopy. Cell nuclei were counterstained with DAPI. Representative microphotographs, scale bar—50 µm; (**d**) Evaluation of HIF-1α nuclear translocation, based on the nuclear-to-cytoplasmic fluorescence intensity ratio (N/C ratio), calculated using CellProfiler software. Data are presented as mean ± SD, **** *p* < 0.0001; CBX—carbenoxolone, a specific gap junction inhibitor.

Analysis by quantitative real-time PCR (qRT-PCR) showed a significant increase in *HIF1A* expression levels in cells subjected to hypoxic exposure (<1% O_2_, 24 h) compared to the control (20% O_2_) by 5-fold (EA.hy926 cells) and 8-fold (MSCs) (*p* ≤ 0.05), indicating activation of the hypoxic response at the transcriptional level (Figure 1a).

An increase in HIF-1α levels was also observed at the protein level using flow cytometry (Figure 1b). The mean fluorescence intensity (MFI) increased significantly by 1.4-fold (EA.hy926 cells) and 1.7-fold (MSCs) in the samples compared to normoxic conditions (*p* ≤ 0.05).

Blockade of GJs with the specific inhibitor carbenoxolone (CBX) significantly reduced HIF-1α, both at the transcription level and at the protein level, compared to cells exposed only to hypoxia. At the expression level, *HIF1A* decreased 1.2-fold in EA.hy926 cells and 2.3-fold (*p* ≤ 0.05) in MSCs, and at the protein level, it decreased 1.9-fold in EA.hy926 cells and 2.3-fold in MSCs (*p* ≤ 0.05) (Figure 1a,b).

Additionally, HIF-1α immunofluorescent staining was performed and visualized using a Zeiss LSM 900 confocal microscope (Carl Zeiss, Jena, Germany) (Figure 1c and Figure S1). Quantitative image analysis was conducted to determine HIF-1α distribution based on the mean fluorescence intensity within the nuclear and cytoplasmic compartments. Under hypoxic conditions, a marked enhancement of nuclear fluorescence was observed, consistent with the nuclear translocation of stabilized HIF-1α. Quantitative assessment of the nuclear-to-cytoplasmic fluorescence intensity ratio (N/C) demonstrated a 23% increase following hypoxic exposure (Figure 1d). Notably, CBX treatment did not induce any significant alterations in HIF-1α translocation under hypoxic conditions.

### 2.2. Gap Junctions During Hypoxic Stress

**Figure 2:**
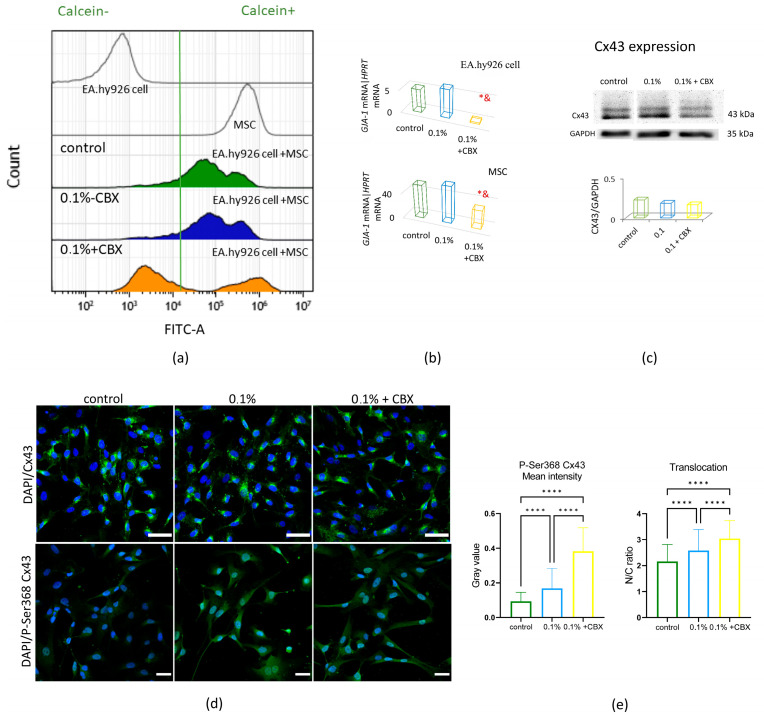
Assessment of the efficiency of intercellular communication between MSC- EA.hy926 cell via gap junctions: (**a**) Representative histograms. “Parachute” assay, flow cytometry. Distribution of cells based on mean fluorescence intensity (MFI): non-labeled EA.hy926 cells suspension (peak 1); suspension of calcein-labeled MSCs before co-culture (peak 2); co-culture distribution after interaction between calcein-loaded MSCs and non-labeled EA.hy926 cells at control (20% O_2_) (peak 3); co-culture distribution after interaction between calcein-loaded MSCs and non-labeled EA.hy926 cells at 0.1% O_2_ (peak 4); co-culture distribution after interaction between calcein-loaded MSCs and non-labeled EA.hy926 cells at 0.1% O_2_ in the presence of gap junction blocker (peak 5); (**b**) Transcriptional activity of the *GJA1* gene encoding the gap junction protein connexin 43 (Cx43) in EA.hy926 cells and MSCs. Data are presented as mean ± SD, *n* = 4. * *p* ≤ 0.05 vs. control (20% O_2_); & *p* ≤ 0.05 vs. 0.1% O_2_; (**c**) Representative Western Blot and quantitative analysis of Cx43 expression in co-culture cells. Band intensities were normalized to GAPDH; (**d**) Immunofluorescent staining of Cx43 and phosphorylated Cx43 at the Ser368 site (P-Ser368 Cx43) in co-culture cells analyzed by confocal microscopy. Cell nuclei were counterstained with DAPI. Representative microphotographs, scale bar—100 μm; (**e**) Quantitative assessment of P-Ser368 Cx43 staining intensity in co-culture cells and evaluation of its nuclear translocation were performed based on the nuclear-to-cytoplasmic fluorescence intensity ratio (N/C ratio). Image analysis was conducting using CellProfiler software. Data are presented as mean ± SD, **** *p* < 0.0001; CBX—carbenoxolone, a specific gap junction inhibitor.

The functional activity of GJs between MSCs and EA.hy926 cells was confirmed by parachute assay using flow cytometry. After 3 h of co-culture of calcein-stained MSCs with unstained EA.hy926 cells (ratio 30 to 70%), a single cell population with normal fluorescence distribution was recorded, where the average fluorescence intensity (MFI) was 127 ± 15 × 10^3^ relative units at control (20% O_2_) (peak 3) and 161 ± 26 × 10^3^ relative units at 0.1% O_2_ (peak 4) (Figure 2a). These values were significantly higher than in the initial unstained EA.hy926 cells (peak 1) (0.5 ± 0 × 10^3^ rel. units), but lower than in stained MSCs (peak 2) (5395 ± 620 × 10^3^ rel. units), indicating active transfer of calcein through gap junctions (Figure 2a).

Upon addition of CBX under hypoxic conditions, calcein transfer was practically absent (Figure 2a). The histograms of MFI distribution in this case showed a biphasic curve (a two-peak curve): MSCs stained with CBX maintained a high level of fluorescence (MFI 806 ± 189 × 10^3^ rel. units), while EA.hy926 cells had an extremely low level of fluorescence (MFI 5 ± 0 × 10^3^ rel. units) (peak 5) (Figure 2a). This indicates an effective inhibition of intercellular calcein transfer when exposed to CBX.

Analysis of the expression of the *GJA1* gene encoding connexin 43 (Cx43), the main connexin protein forming GJs, showed a 1.2-fold increase in mRNA levels in MSCs after hypoxic exposure compared to normoxia (Figure 2b). At the same time, the use of CBX led to a significant decrease in *GJA1* expression: 2-fold (in EA.hy926 cells ) and 19-fold (in MSCs) (*p* ≤ 0.05) (Figure 2b).

Semiquantitative analysis confirmed a reduction in Cx43 expression following channel blockade (Figure 2c). In immunofluorescent staining, Cx43 was predominantly localized at the plasma membrane, consistent with its role in the formation of intercellular junctions (Figure 2d). Evidence of gap junction dysfunction after CBX treatment was further supported by an increase in phosphorylated Cx43 at the Ser368 site. In parallel, exposure to hypoxia, both alone or in combination with CBX, induced additional Cx43 phosphorylation and promoted its redistribution toward the nuclear region (Figure 2e).

### 2.3. Viability

Viability analysis showed that under conditions of hypoxia alone, the proportion of viable cells remained high: 93% in the MSCs population and 91% among EA.hy926 cells (Table 1). However, a moderate increase in the level of apoptosis and necrosis was recorded (Table 1). More pronounced changes were observed under the combined effects of hypoxia and GJ blockade (Table 1).

### 2.4. Proliferative Activity of Cells Under Hypoxic Stress and Impaired Intercellular Interactions

The proliferative activity of MSCs and EA.hy926 cells was analyzed using flow cytometry (assessment of cell cycle phase distribution) and the WST assay (assessment of metabolic activity as an indirect marker of proliferation).

**Figure 3:**
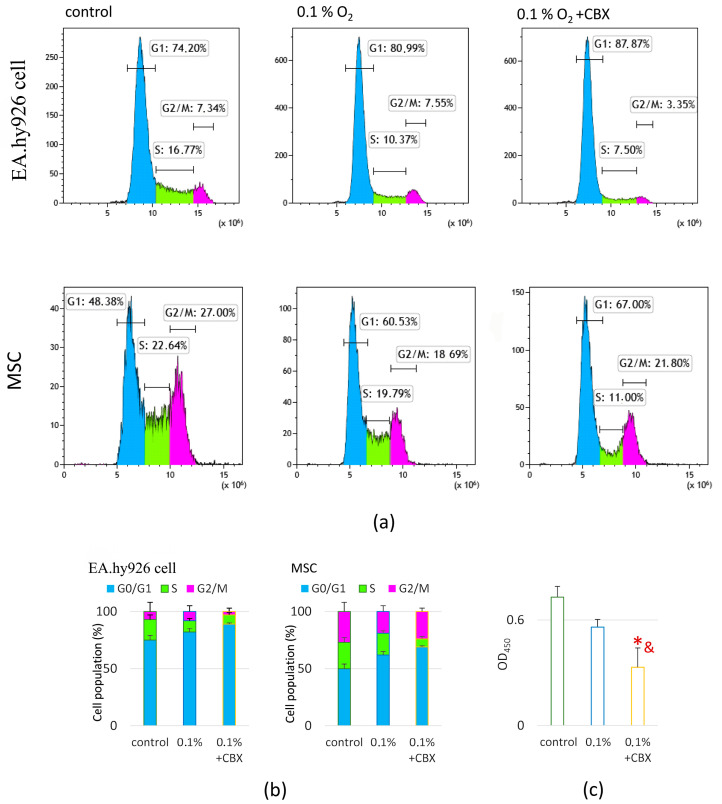
Effect of combined carbenoxolone and hypoxia treatment on the proliferative activity of MSC-EA.hy926 cell co-culture: (**a**) Cell cycle analysis by flow cytometry after Hoechst staining; representative histograms are shown; (**b**) Quantification of mean fluorescence intensity in MSCs and EA.hy926 cells across different cell cycle phases: G_1_/G_0_ (growth and resting phase), S (DNA synthesis phase), and G_2_/M (preparation for mitosis/mitotic phase); (**c**) Colorimetric analysis of metabolically active cells using the Cell Proliferation Reagent WST-1 (WST assay). Data are presented as mean ± SD, *n* = 4. * *p* ≤ 0.05 vs. control (20% O_2_); & *p* ≤ 0.05 vs. 0.1% O_2_. CBX—carbenoxolone, a specific gap junction inhibitor.

Under acute hypoxic stress (0.1% O_2_), changes in the distribution of cells by cell cycle phases were observed in EA.hy926 cells and MSCs populations (Figure 3a). In EA.hy926 cells, there was no significant decrease in the proportion of cells in the G_2_/M phase, but there was a 1.6-fold decrease in the number of cells in the S phase compared to the control (20% O_2_) (Figure 3b). At the same time, in MSCs, hypoxia caused a 1.4-fold decrease in the proportion of cells in the G_2_/M phase, accompanied by an increase in the number of cells in the G_1_ phase (Figure 3b). Such a redistribution may indicate a partial delay in the cell cycle in the early stages of proliferation under the influence of hypoxic stress.

Under the combined effect of hypoxia and GJ blockade, more pronounced changes in the proliferative status of both cell types were observed (Figure 3a,b). In EA.hy926 cells, the proportion of cells in the G2/M phase decreased by twofold, mainly due to an increase in the number of cells in the G_1_ phase (Figure 3b). In the MSC population, under the influence of CBX, the proportion of cells in G2/M decreased by 1.2-fold, and the number of cells in the S phase decreased by 2.9-fold (Figure 3b). At the same time, an increase in the proportion of cells in the G1 phase was noted, which may indicate a pronounced blockage of the cell cycle at an early stage.

Under hypoxic conditions, a tendency toward a decrease in metabolic activity was observed. Under the combined effects of hypoxia and GJ inhibition, a more pronounced decrease in metabolically active cells was observed, 2.2-fold compared to the control (20% O_2_) and 1.7-fold compared to the effect of hypoxia, which correlated with the cell cycle analysis data (*p* ≤ 0.05) (Figure 3c).

### 2.5. Cell Migration Activity Under Hypoxic Stress and Gap Junction Blockade

Cell migration properties were assessed in two experimental models: random migration was assessed in vitro using the wound healing assay (scratch assay) and directed migration using decellularized extracellular matrix (dcECM) from MSCs as a chemoattractant stimulus.

**Figure 4:**
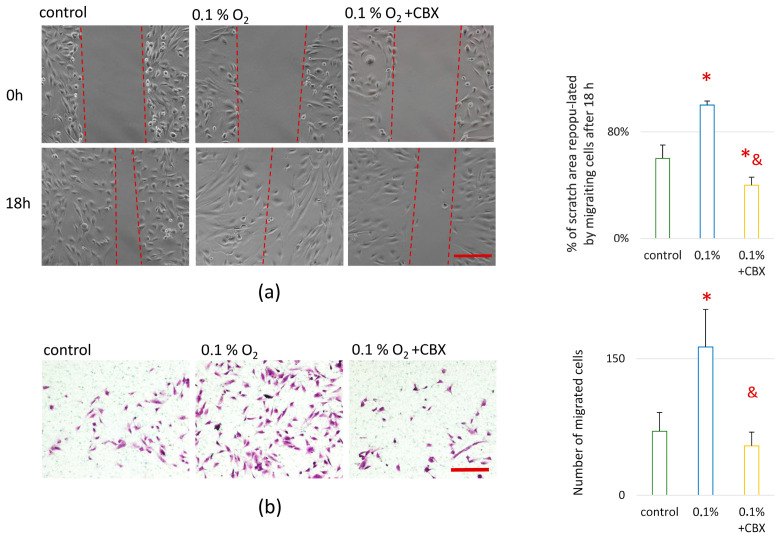
Effect of combined carbenoxolone and hypoxia treatment on migration in MSC-EA.hy926 cell co-culture: (**a**) Random migration in wound healing assay. Representative microphotographs (light microscopy) and gap closure (%) after 18 h. The gap closure was calculated as (1 − N/N0) × 100%, where N-final wound area (18 h after scraching) and N0-initial wound area (0 h after scraching). Scale bar—200 μm, objective: 10× lens. Red dashed lines indicate the wound width in the scratch assay images; (**b**) Directed migration of MSC-EA.hy926 cell co-culture toward decellularized extracellular matrix (dcECM) used as a chemoattractant stimulus. Scale bar—500 μm, objective: 4× lens; Data are presented as mean ± SD, *n* = 4. * *p* ≤ 0.05 vs. control (20% O_2_); & *p* ≤ 0.05 vs. 0.1% O_2_. CBX—carbenoxolone, a specific gap junction inhibitor.

Under hypoxic stress (0.1% O_2_), increased migration activity was observed in both models. Thus, directed cell migration toward dcECM increased 2.3-fold, while random migration increased 1.6-fold compared to the control (20% O_2_) (Figure 4a,b). These results indicate the stimulating effect of hypoxia on the ability of cells to move both in response to chemotactic signals and under conditions of spontaneous restoration of the damaged monolayer in culture.

The combined effect of hypoxia and GJ blockade led to a marked change in cell migration. In particular, under conditions of dual exposure (hypoxia + CBX action), random migration decreased by 1.5-fold compared to hypoxia, which was statistically significant (*p* ≤ 0.05) (Figure 4a). At the same time, in the model of directed migration to the dcECM, the changes were leveled out (Figure 4b): the values did not differ from the control (20% O_2_), which indicates a less pronounced dependence of spontaneous cell migration on gap intercellular interactions.

### 2.6. Angiogenic Potential of Associates

The next stage of our study was aimed at a comprehensive assessment of the angiogenic potential generated by MSC-EA.hy926 cell associates. It is known that paracrine secretion plays a key role in the regulation of physiological angiogenesis, affecting the activation, migration, and proliferation of ECs [27,28]. Under conditions of hypoxia and impaired GJ function, intercellular communication changes significantly, which may affect the angiogenic profile of cell cultures. To assess these changes, the conditioned medium (CM) obtained after co-culturing cells was analyzed. To understand the role of functionally active GJs in the angiogenic potential of the secretome obtained after 24 h of interaction between MSCs and EA.hy926 cells under hypoxic stress (0.1% O_2_), a series of in vitro and in ovo functional tests were performed. CM was used to analyze the ability to induce key stages of angiogenesis: cell migration, tubular structure formation, and stimulation of vascular network development in the chorialallantoic membrane (CAM) model of Japanese quail (*Coturnix coturnix japonica*) eggs.

#### 2.6.1. Secretory Profile

**Figure 5:**
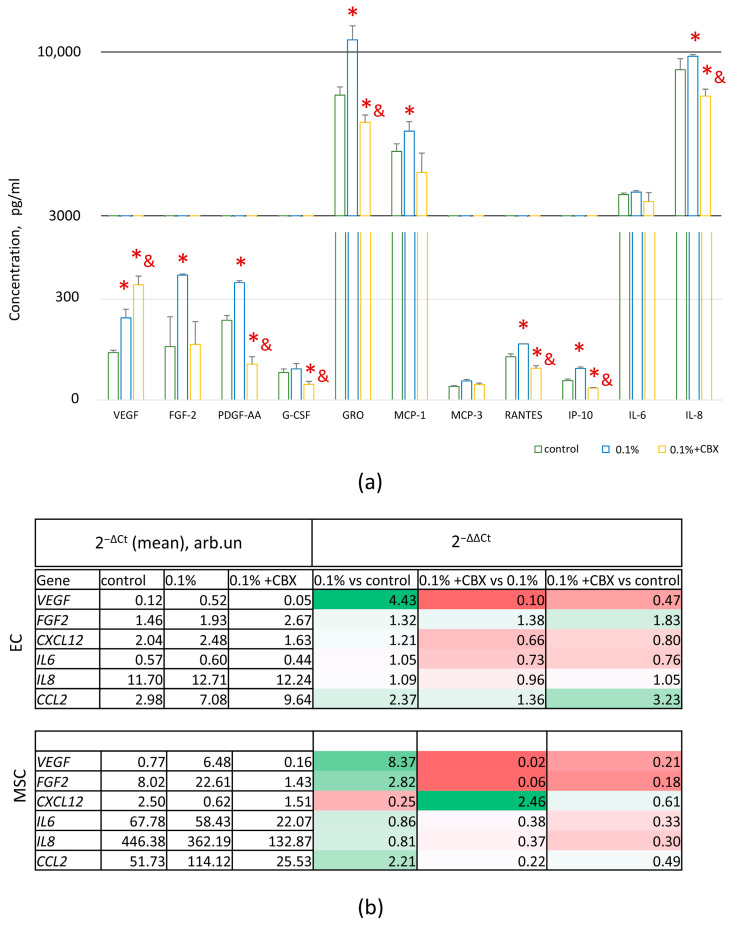
Secretome-associated gene expression and cytokine profiling in MSC-EA.hy926 cell co-culture: (**a**) Differential gene expression related to the secretome in EA.hy926 cells and MSCs after co-culture. Normalized gene expression was calculated using the 2^−ΔCt^ and 2^−ΔΔCt^ methods. Red indicates significantly upregulated genes, green—significantly downregulated genes, and transparent—no significant changes (*p* ≤ 0.05); (**b**) Cytokine levels in the conditioned medium collected after co-culture. Data are presented as mean ± SD, *n* = 4. * *p* ≤ 0.05 vs. control (20% O_2_); & *p* ≤ 0.05 vs. 0.1% O_2_. CBX—carbenoxolone, a specific gap junction inhibitor.

The results showed that under hypoxia (0.1% O_2_) in CM, the content of key angiogenic factors significantly increased: VEGF (1.7-fold), FGF-2 (2.3-fold), PDGF-AA (1.5-fold), GRO (1.3-fold), MCP-1 (1.2-fold), RANTES (1.3-fold), IP-10 (1.6-fold), and IL-8 (1.2-fold) (*p* ≤ 0.05) (Figure 5a). This indicates the activation of the angiogenic response as a result of hypoxic exposure. Interestingly, against the background of hypoxia, a significant decrease in G-CSF levels was observed compared to control (20% O_2_), suggesting selective suppression of certain links in the cytokine response. However, when the GJ was blocked, these effects were neutralized, with the exception of VEGF, whose level continued to increase (1.4-fold compared to the effect of hypoxia) (*p* ≤ 0.05) (Figure 5a).

Gene expression analysis revealed a similar pattern: most of the angiogenic factors we studied showed an increase in mRNA levels at 0.1% O_2_, followed by a decrease when GJ was blocked (Figure 5b). The exceptions were IL-6 and IL-8, whose expression did not increase under hypoxia but decreased when intercellular communication was inhibited. At the same time, VEGF showed a steady increase at 0.1% O_2_, but when GJs were blocked, their expression decreased despite the increased protein level in the environment (Figure 5a,b).

The dynamics of *CXCL12* chemokine expression under hypoxic stress is of interest, as it plays a key role in angiogenesis, cell migration, and maintenance of the microenvironment of ECs [29,30]. At 0.1% O_2_ in MSCs, the level of *CXCL12* expression decreased more than 4-fold (*p* ≤ 0.05) (Figure 5b). At the same time, in EA.hy926 cells, expression remained stable under hypoxia but decreased with additional GJ blockade (Figure 5b). In contrast, in MSCs, blocking intercellular communication against a background of hypoxia led to the restoration of *CXCL12* expression to the control level (20% O_2_) (Figure 5b).

#### 2.6.2. Random Migration of EA.hy926 Cells

**Figure 6:**
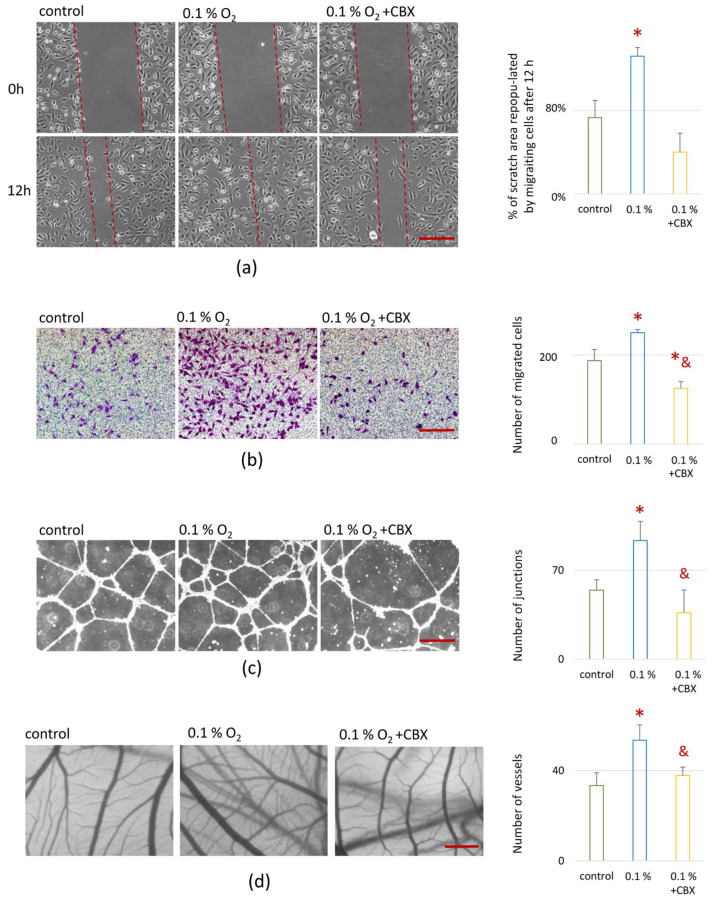
Effect of conditioned medium from MSC-EA.hy926 cell co-culture on EA.hy926 cell migration and angiogenic activity in vitro and in ovo: (**a**) EA.hy926 cells’ random migration in wound healing assay. Representative microphotographs (light microscopy) and gap closure (%) after 12 h. The gap closure was calculated as (1 − N/N0) × 100%, where N is final wound area (12 h after scraching) and N0 is initial wound area (0 h after scraching). Scale bar: 200 μm, objective: 10× lens. Red dashed lines indicate the wound width in the scratch assay images; (**b**) Directed migration of EA.hy926 cells toward decellularized extracellular matrix (dcECM) and conditioned medium used as a chemoattractant stimulus. Scale bar: 500 μm, objective: 4× lens; (**c**) The capillary-like tube formation by EA.hy926 cells in vitro. Representative images of the capillary-like tubes in “Matrigel” (light microscopy) and number of tubule complexes. Scale bar: 200 μm, objective: 10× lens; (**d**) Angiogenesis in ovo. Representative images of chorioallantoic membrane (CAM) blood vessel network staining (light microscopy) and vessel number. Light field, scale bar: 2 mm, 60× magnification; Data are presented as mean ± SD, *n* = 4. * *p* ≤ 0.05 vs. control (20% O_2_); & *p* ≤ 0.05 vs. 0.1% O_2_. CBX—carbenoxolone, a specific gap junction inhibitor.

When applying CM from MSC-EA.hy926 cell 0.1% O_2_, random EA.hy926 cell migration increased 1.3-fold compared to the control (20% O_2_) (Figure 6a). This indicates a significant activation of overall cell motility. When a GJ blocker was added to the co-culture under hypoxic conditions, this effect was completely negated: migration decreased to the control level (20% O_2_). 

#### 2.6.3. Directed Migration of EA.hy926 Cells

CM collected after 24 h of cell culture at 0.1% O_2_ caused a significant increase in directed EA.hy926 cell migration by 30% compared to the control medium (20% O_2_), indicating the activation of chemotactic mechanisms under hypoxic conditions (Figure 6b). Under conditions of GJ blockade, the migration level not only returned to control values (20% O_2_), but also decreased significantly below baseline, which may indicate suppression of endothelial activation in the absence of intercellular signal transmission.

#### 2.6.4. Tube Formation Assay in Matrigel

The formation of capillary-like structures in Matrigel under the action of CM collected in conditions of 0.1% O_2_ increased twofold compared to the control (20% O_2_) (Figure 6c). A significant increase in the number of tubular structures was observed, demonstrating active angiogenesis in vitro. The addition of a GJ blocker completely eliminated this effect: the number of structures corresponded to the control group (20% O_2_), with no signs of increased tubulogenesis (Figure 6c).

#### 2.6.5. Chorionallantoic Membrane Assay (CAM Assay)

In order to assess how CM from co-culture would affect the growth and development of the actual vascular network, we used an in ovo method that allows characterizing the formation of the vascular network in the CAM of a quail embryo. CM obtained after hypoxic exposure caused a significant increase in the number of newly formed vessels by more than twofold (*p* ≤ 0.05) compared to the control (20% O_2_) (Figure 6d). CM collected from cells after blocking the GJs was accompanied by an absence of vascular growth, with a return to the control level (20% O_2_) (Figure 6d).

## 3. Discussion

Based on the obtained results, it can be concluded that GJs, mediated mainly by connexin 43 (Cx43), play a critical role in ensuring a coordinated angiogenic response between MSCs and EA.hy926 (an Endothelial Cell Model), especially under conditions of acute hypoxic stress. The maintenance of functioning GJs contributed to the preservation of cell proliferation, migration, and secretion of angiogenic factors, while their blockade led to a marked suppression of all these parameters.

One of the key molecular mediators of the hypoxic response is the transcription factor HIF-1α, which regulates the expression of more than 2% of genes, including proangiogenic factors [31,32,33,34,35,36,37]. Our data showed that blocking GJs weakened HIF-1α activation: its expression decreased and nuclear translocation was disrupted, confirming the role of intercellular communication in the stability of the hypoxic response. This may be due to the possible synchronization of signaling cascades via the GJs, including the transmission of Ca^2+^ ions, cAMP, and other second messengers [8]. Under hypoxic conditions, cells often modulate GJs by altering connexin expression, a response that affects the distribution of metabolic substrates between neighboring cells. Early studies on astrocytes have shown that hypoxia induces an increase in the expression of Cx43, the main protein of GJs, contributing to the regulation of metabolic exchange, in particular lactate and glucose, to maintain cell viability [38]. Similar effects have since been observed in other cell types, including fibroblasts, where preservation of GJs during chronic hypoxia helped maintain ATP levels [39].

Our results did not reveal any changes in Cx43 expression after 24 h of hypoxic exposure in MSC- EA.hy926 co-culture; however, a decrease in its expression was observed under the combined effect of CBX and hypoxia. An effect on its phosphorylated form at the Ser368 site was also observed. Phosphorylation of Cx43 at serine 368 is a well-known and important post-translational modification involved in the regulation of gap junctions, particularly their permeability and intercellular communication [40]. Ser368 phosphorylation promotes channel closure, internalization, and degradation of Cx43 via proteasome and lysosomal pathways, thereby reducing Cx43 levels at the plasma membrane and contributing to decreased communication through GJs, especially under stress or pathological conditions [41]. Enhanced modification of Cx43 during hypoxia may indicate activation of the cellular stress response. When CBX was added, an increase in P-Cx43 fluorescence intensity and its intracellular redistribution toward the nuclear region were observed. This suggests a direct effect of CBX on Cx43 phosphorylation, accompanied by stimulation of its intracellular degradation. Since modulation of Cx43 phosphorylation status affects cellular adaptations, including those associated with hypoxia, specific molecular cross-interactions between pSer368 Cx43 levels and the activation of HIF-1α-dependent pathways require further investigation.

Analysis of cell distribution by cell cycle phases showed that hypoxia induces partial cell cycle arrest predominantly at the G_1_ phase, which is consistent with literature data on the suppression of G_1_/S transition under hypoxic conditions [42,43]. A decrease in the proportion of cells in the S phase with a simultaneous increase in the G_1_ phase may indicate metabolic overload of cells and an adaptation to reduced oxygen and energy availability. When GJs were blocked, these effects were amplified: proliferative activity decreased more significantly, especially in EA.hy926 cells, which showed a sharp reduction in the number of cells in the S phase and G_2_/M, which may indicate inhibition not only of G_1_ exit but also of mitosis completion. Such changes may be associated with impaired intercellular signal coordination, including calcium regulation, since GJs (particularly Cx43) mediate the transmission of Ca^2+^ and other signaling molecules that promote synchronous cell cycle progression [8,44]. Under hypoxic conditions, Ca^2+^ plays a key role in activating the CaMK2 → TAK1 → IKK → NF-κB signaling cascade, which may ensure cell survival and trigger adaptive programs [45]. Blocking the GJs can disrupt this pathway, preventing NF-κB activation and, as a result, reducing the transcriptional activity of proliferative genes. Metabolic activity, as an indirect marker of proliferation, supports these findings: the combination of hypoxia and GJs blockade resulted in a marked suppression of metabolism, which correlates with cell cycle data.

Similar patterns were observed when assessing the migratory activity of co-culture: we observed an increase in both directed and spontaneous migration under hypoxia condition, which is consistent with studies showing the activation of not only HIF-dependent mechanisms, but also NF-κB, MAPK/ERK, and PI3K/AKT signaling pathways involved in the regulation of cell motility and adaptation to oxygen deficiency [46,47,48,49]. Blocking GJs neutralized the stimulating effect of hypoxia on migration activity. At the same time, the greatest decrease was observed precisely in conditions of random migration, where the values fell below both hypoxic and control values. One possible mechanism for suppressing migratory activity when GJs are blocked is the disruption of intercellular coordination. GJs facilitate the exchange of signaling molecules, including calcium ions, cAMP, and IP_3_, which is critical for synchronizing the movement of cell populations. In addition, as mentioned earlier, blocking GJs can suppress the activation of the calcium-dependent signaling cascade CaMK2-TAK1-IKK-NF-κB, which plays an important role in cellular adaptation to hypoxic stress [45]. Activation of this pathway is typically accompanied by the expression of genes involved in cytoskeletal regulation, metalloproteinase (MMP) production, and changes in cell adhesion and migration. In the absence of calcium signaling between cells, these adaptive mechanisms may be disrupted, leading to a decrease in migratory potential despite the presence of a hypoxic stimulus. Equally important is the fact that GJs are involved in the spatial polarization of cells, which is necessary for the formation of directed movement. It is known that connexin-dependent signals affect the activity of small GTPases (Rac1, Cdc42), focal adhesions kinase (FAK), and actin cytoskeleton reorganization [50,51,52,53].

Analysis of the angiogenic activity of MSC-EA.hy926 cell associates confirmed that functional GJs play a crucial role in regulating mediator secretion to ensure a functional angiogenic response. Under hypoxic condition, the production of VEGF, FGF-2, GRO, and PDGF-AA increased, indicating the activation of factors involved in the regulation of the vascular network. However, when the GJs were inhibited, a pronounced restructuring of this functional profile was observed. CM from cells with blocked GJ lost its ability to stimulate migration, tubule formation, and angiogenesis in CAM, despite increased levels of the key angiogenic factor VEGF in the environment. The increase in VEGF levels after hypoxic exposure combined with GJ blockade may be due to a decrease in VEGF utilization by the endothelium. VEGF actively binds to high-affinity tyrosine kinase receptors VEGFR-2 (KDR/Flk-1) on the surface of EC, after which it undergoes endocytosis and metabolic processing, which regulates its availability in the microenvironment [54,55]. Disruption of communication via the GJs could have impeded adequate receptor activity and coordinated VEGF uptake, consistent with the role of GJs in organizing intracellular receptor trafficking and modulation sensitivity to growth factors [50,53]. This could be due to both impaired cooperation between MSCs and endothelial-like cells and a reduction in the complex of factors necessary for a full angiogenic signal.

An interesting finding was the difference in the degree of reduction in the angiogenic response depending on the type of migration studied. More pronounced suppression was observed under conditions of directed migration compared to random migration. An increase in VEGF levels with a simultaneous decrease in other growth factors, such as IL-8 and PDGF-AA, may indeed enhance directed cell migration and weaken undirected migration. VEGF predominantly activates directed chemotaxis by stimulating interaction with high-affinity VEGFR-2 receptors, which leads to the activation of signaling pathways that regulate EC migration and angiogenesis. This ensured the formation of a directed migratory response of cells in response to VEGF gradients [54,55]. At the same time, random migration depends to a greater extent on overall cell tone, proliferative activity, and less specific stimuli such as FGF-2, IL-8, and PDGF-AA, which affect the cytoskeleton and adhesion substrates [56,57]. A reduction in these factors could weaken these processes, reducing the basal migratory activity of cells outside of directed gradients. Similar discrepancies between VEGF stimulation and the actual angiogenic effect were also reflected in the tubular structure formation test in Matrigel and the CAM angiogenesis model. This could be due not only to a decrease in the concentrations of proangiogenic factors, but also to a potential increase in the level of antiangiogenic molecules, which further inhibits vascular growth.

Thus, GJs not only provide structural connections between cells, but also play an active functional role in the formation of a coordinated angiogenic response under hypoxic conditions. Their involvement in the regulation of key signaling pathways, stress adaptation, and secretion profile control makes them a promising target in the development of new approaches to restore ng microcirculation in ischemic and inflammatory damage.

## 4. Materials and Methods

### 4.1. Cell Culture and Experimental Design

**Figure 7:**
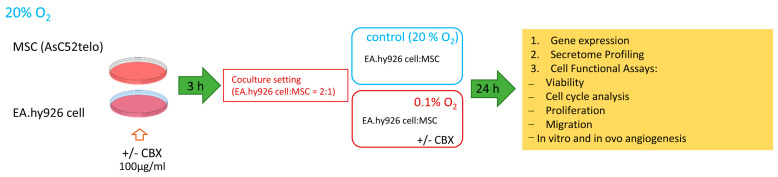
Schematic representation of the experimental workflow. CBX—carbenoxolone, a specific gap junction inhibitor.

The study used linear MSCs (ASC52telo, ATCC^®^ SCRC-4000™, Manassas, VA, USA); and endothelial cell model ECs (EA.hy926, ATCC^®^ CRL-2922™, Manassas, VA, USA) obtained on a non-commercial basis from a cell repository as part of the Noah’s Ark project at Lomonosov Moscow State University. The EA.hy926 cell line (a hybrid of human endothelial cells and A549 lung adenocarcinoma cells) was used to model ECs. This line is widely used as an endothelial cell model; however, it is not a primary EC, which should be taken into account when interpreting the results. The cells were cultured in DMEM/F12 medium (Gibco, Life Technologies, Carlsbad, CA, USA) supplemented with 10% fetal calf serum HyClone (Cytiva, Marlborough, MA, USA) and 100 IU/mL penicillin/100 μg/mL streptomycin (Panecho, Moscow, Russia). After 48 h, a specific inhibitor, carbenoxolone (CBX) (Sigma-Aldrich, St. Louis, MO, USA), at a concentration of 100 μg/mL was added to part of the MSCs and EA.hy926 cells to block GJs. After 3 h, the cells were washed from the inhibitor, trypsinized, and dispersed into Petri dishes in a 2:1 ratio (EA.hy926 cell:MSC). The co-culture was cultivated under standard laboratory conditions in a CO_2_ incubator (Sanyo, Osaka, Japan) and subjected to hypoxic exposure to 0.1% O_2_ in a hypoxic chamber (STEMCELL Technologies, Vancouver, BC, Canada) equipped with an oxygen sensor for 24 h (Figure 7). Upon completion of the exposure, both the cell cultures and the collected conditioned medium (CM) were analyzed.

### 4.2. Assessment of Gap Junctional Intercellular Communication

Communication via GJs was assessed using the fluorescent probe Calcein AM (Invitrogen, Carlsbad, CA, USA), a derivative of which, calcein, has been shown to selectively transfer from cell to cell via GJs. Calcein fluorescence was detected in the FITC channel. Suspensions of Calcein AM-stained MSCs and unstained EA.hy926 cells were mixed at a 1:2 ratio and co-cultured for 3 h (parachute assay) [9,58]. MSCs were labeled with Calcein AM at a concentration of 2 µM for 30 min at 37 °C. Following incubation, cells were washed three times with fresh medium to remove extracellular or unincorporated dye prior to co-culture with EA.hy926 cells, in accordance with the manufacturer’s protocol. After incubation, the heterocultures were detached from the plastic using trypsin-EDTA (HiMedia, Mumbai, India) for subsequent analysis by flow cytometry (CytoFLEX, Beckman Coulter Life Sciences, Indianapolis, IN, USA). The co-culture suspensions were stained with anti-CD90-PB450 antibodies (Beckman Coulter Life Sciences, USA) against Thy-1, which is expressed exclusively by MSCs, and anti-CD31-PE antibodies (Beckman Coulter Life Sciences, Indianapolis, IN, USA) targeting the endothelial marker PECAM-1. This staining allowed identification of the respective cell populations via flow cytometry and detection of dye transfer to EA.hy926 cells (CD31+/CD90−). Dye transfer to EA.hy926 cells was confirmed by the appearance of a fluorescent EA.hy926 cell population in the corresponding channel. For accurate flow cytometry analysis, both single-stained controls for each fluorophore and an unstained control were used. Single-stained controls allowed for compensation of spectral overlap between Calcein AM, CD90-PB450, and CD31-PE, while the unstained control was used to assess the background autofluorescence of the cells.

### 4.3. Immunofluorescence Staining

Cells were washed twice with phosphate-buffered saline (PBS) (Panecho, Moscow, Russia) and fixed with 4% paraformaldehyde at 37 °C for 15 min. For permeabilization, cells were incubated with 0.2% Triton X-100 for 10 min at room temperature. Nonspecific antibody binding was blocked with 3% bovine serum albumin (BSA, Sigma, USA) for 30 min at room temperature. Cells were then incubated with the following primary rabbit anti-human antibodies at +4 °C in a humidified chamber overnight: Anti-connexin43 (ab217676, 1:500; Abcam, Cambridge, UK ), Anti-connexin43 phospho S368 (ab194928, 1:500; Abcam, Cambridge, UK), and Anti-HIF1α (E-AB-31662, 1:100; Elabscience, Wuhan, China). Detection of the primary antibodies was performed using goat anti-rabbit IgG–FITC conjugated secondary antibody (E-AB-1014, 1:100, Elabscience, Wuhan, China), incubated for 2 h at room temperature. Slides were mounted using Fluoroshield mounting medium containing DAPI (Sigma-Aldrich, St. Louis, MO, USA). As a negative control, cells were incubated with the secondary antibody only (without primary antibodies). Fluorescence images were acquired using a Zeiss LSM 900 confocal microscope (Carl Zeiss, Jena, Germany).

Quantitative image analysis was performed using CellProfiler software (v.4.2.6). For each experimental condition, approximately 500 cells were analyzed, derived from three independent biological replicates (separate experiments). Nuclei were segmented from the DAPI channel using they IdentifyPrimaryObjectsmodule. Based on the identified nuclei, whole-cell segmentation was performed on FITC channel using the IdentifySecondaryObjects module, generating regions of interest (ROIs) corresponding to individual cells. The mean fluorescence intensity (MFI) was measured within these ROIs. Nuclear translocation was assessed by calculating the nuclear-to-cytoplasmic (N/C) fluorescence intensity ratio. After segmenting nuclei and cells, the cytoplasmic ROI was obtained by subtracting the nuclear ROI from the whole-cell ROI. The mean fluorescence intensity was measured separately within the nuclear and cytoplasmic regions. The N/C ratio was calculated as: N/C Ratio = MFI_nucleus/MFI_cytoplasm. An increase in the N/C ratio was interpreted as enhanced translocation into the nucleus.

### 4.4. Immunofluorescence Staining of Cells to Detect HIF-1α

After cultivation, the cells were washed with PBS and treated with Accutase (STEMCELL Technologies, Vancouver, BC, Canada). The resulting suspension was fixed with 4% paraformaldehyde, then washed with PBS (Panecho, Moscow, Russia) and methanol was added. After centrifugation, the cells were incubated with anti-CD90-PB450 antibodies (Beckman Coulter Life Sciences, Indianapolis, IN, USA), then washed. The samples were divided into three groups: (1) negative control (CD90), (2) secondary antibody control (CD90 + FITC), (3) experiment (CD90 + HIF-1α + FITC) (Figure S2). To detect HIF-1α, cells were incubated with anti-HIF-1α primary antibody (Elabscience, Wuhan, China), washed, and stained with anti-rabbit IgG-FITC secondary antibody (Elabscience, Wuhan, China). After final washing, the cells were resuspended in PBS (Panecho, Moscow, Russia) and analyzed on a flow cytometer.

### 4.5. Western Blot Analysis

Cells were lysed in RIPA buffer supplemented with protease and phosphatase inhibitors (Elabscience, Wuhan, China). Protein concentrations were determined using the BCA protein assay (Thermo Fisher Scientific, Waltham, MA, USA). A total of 10 µg of protein per sample was separated on 12.5% SDS-polyacrylamide gels. For molecular weight reference, PageRuler™ Prestained Protein Ladder (Thermo Fisher Scientific, Waltham, MA, USA) was used.

Proteins were electro-transferred to nitrocellulose membranes using a wet transfer method in buffer composed of 25 mM Tris, 190 mM glycine, 20% methanol, pH 8.3 at 80 mA for 12 h at +4 °C. Transfer efficiency was confirmed by Ponceau S staining (Figure S3). Membranes were blocked for 1 h at room temperature in 4% BSA in 0.1% PBS-T and incubated with primary antibodies for 2 h at room temperature. Cx43 (1:1000; Abcam, ab217676, Cambridge, UK); GAPDH (1:5000; Elabscience, E-AB-40337, Wuhan, China). After washing, membranes were incubated with horseradish peroxidase-conjugated secondary antibodies ((1:5000; Elabscience, E-AB-1003, Wuhan, China) for 2 h at room temperature. Chemiluminescent signals using Super Excellent Chemiluminescent Substrate (ECL) Diagnostic Kit (Elabscience, Wuhan, China) were detected using Biorad ChemiDoc digital imaging system (Bio-Rad, Hercules, CA, USA) with exposure times adjusted to ensure all signals were within the linear detection range. Densitometric analysis was performed using Image Lab software 5.0 (Bio-Rad, Hercules, CA, USA). The data obtained from a single biological replicate, which represents a limitation of the study.

### 4.6. Cell Viability Assay

Cell viability was determined in the experiment using flow cytometry on a CytoFLEX instrument (Beckman Coulter, USA) with an Annexin V-FITC/PI kit (Immunotech, Marseille, France) according to the manufacturer’s instructions. In the presence of Ca^2+^ and Mg^2+^ ions, annexin binds to phosphatidylserine, which translocate from the inner to the outer leaflet of the plasma membrane during the early stages of apoptosis. Propidium iodide (PI) penetrates the cell and binds to DNA when the membrane is destroyed, and thus the pool of cells that are positively stained for both markers is in the late stage of cell death. The accuracy of our analysis was ensured by using single-stained controls and unstained cells to account for background signal and to perform spectral compensation. For identification of MSCs, anti-CD90-PB450 (Beckman Coulter Life Sciences, Indianapolis, IN, USA) (405 nm laser, 450/45 nm filter) antibody was used. Unstained cells correspond to the EA.hy926 cell population (Figure S4). While Annexin V–FITC (488 nm la-ser, 525/40 nm filter) and PI (488 nm laser, 585/42 nm filter) were applied to assess viability. Fluorophores were chosen with well-separated channels, which minimizes spectral overlap and allows accurate determination of positive events.

### 4.7. Cell Cycle Analysis

The distribution of EA.hy926 cell and MSC populations across cell cycle phases was assessed by flow cytometry using a CytoFLEX instrument (CytoFLEX, Beckman Coulter Life Sciences, Indianapolis, IN, USA) with the fluorescent dye Hoechst 33342 (Thermo Fisher Scientific, Waltham, MA, USA), which binds to DNA and was detected in the PB450 channel (405 nm laser, 450/45 nm filter), according to the manufacturer’s protocol. Hoechst fluorescence intensity correlates with DNA content, allowing the discrimination of cell cycle phases: G0/G1 (low intensity), S (intermediate intensity), and G2/M (double intensity).

For cell type identification, cells were first stained with antibodies against surface markers: MSC—CD90-FITC (Sony, Tokyo, Japan), EA.hy926 cell—CD31-PE (Beckman Coulter Life Sciences, Indianapolis, IN, USA) (Figure S5). Each antibody was used in a separate sample. Surface marker staining was performed prior to fixation to preserve epitope integrity. Cells were then fixed with cold ethanol, which preserves both the surface marker staining and the DNA for accurate Hoechst quantification.

Flow cytometry analysis was performed using a linear scale for Hoechst fluorescence, allowing precise determination of cell cycle phase distribution for each cell population while maintaining accurate identification of MSCs and EA.hy926 cells.

### 4.8. Cell Proliferation Assay

In addition, cell proliferation activity was assessed colorimetrically using the Cell Proliferation Reagent WST-1 kit (Sigma-Aldrich, St. Louis, MO, USA) according to the manufacturer’s instructions. Blanks without cells (containing only medium and WST-1) were included in each experiment to correct for background absorbance and ensure accurate data acquisition. This test is based on the cleavage of water-soluble tetrazolium salt (WST) to formazan by succinate dehydrogenase, which is incorporated into the mitochondrial respiratory chain and is active only in metabolically intact cells. Thus, the amount of formazan formed directly correlates with the number of metabolically active cells in the culture. Sample absorbance was measured using a plate spectrophotometer (Bio-Rad, Hercules, CA, USA) at a wavelength of 450 nm, with correction at a wavelength of 620 nm.

### 4.9. Cell Migration Assays

Random migration (without chemoattractant stimulation) was assessed in vitro using the wound healing assay (scratch assay) based on the average migration area within 6 or 12 h after wounding. The MSC-EA.hy926 cell co-culture subjected to combined hypoxia and GJ blockage and the EA.hy926 cell monocultures treated with CM were studied. Cell motility was assessed by the average migration area (mm^2^), which was the difference between the initial (immediately after wounding) and final wound size (mm^2^). The data obtained were converted into a percentage, taking a completely closed wound as 100%. Area measurements were performed using NIS-elements AR version 3.21 (Nikon, Tokyo, Japan) software.

Directed migration (using decellularized extracellular matrix (dcECM) from MSCs cultured under standard laboratory conditions [59] as a chemoattractant stimulus) was assessed in vitro using the Transwell system (Corning, Corning, NY, USA) [60]. MSC-EA.hy926 cell co-culture or EA.hy926 cell monoculture were added to the Transwell and placed in a 24-well plate with dcECM. When analyzing the directed migration of EA.hy926 cell monoculture, CM served as a secondary chemoattractant stimulus. After 24 h, the cells on the membrane were fixed, stained with crystal violet, photographed, and counted in three fields of view using ImageJ software v2.14.0 (National Institutes of Health, Bethesda, MD, USA).

### 4.10. Capillary-like Tube Formation

The analysis of the ability to form capillary-like structures of EA.hy926 cell was performed using Matrigel (Corning, Corning, NY, USA). Capillary-like structures are defined as closed or branched tubular formations formed by cells on a matrix substrate (Matrigel) and visualized using phase-contrast microscopy. The assessment considered formations at least four cells long, elongated in a single line or branching, connecting to each other in a network, imitating the morphology of the capillary bed. The total number of structures on Matrigel was evaluated in 5 random fields of view in each well using ImageJ software v2.14.0 (National Institutes of Health, Bethesda, MD, USA). To induce the formation of capillary-like structures of EA.hy926 cel on Matrigel, recombinant vascular endothelial growth factor (VEGF) at a concentration of 20 ng/mL was used.

### 4.11. Chorioallantoic Membrane Assay In Ovo

Angiogenic activity of CM was evaluated in chorioallantoic membrane (CAM) assay using Japanese quail embryos. The fertilized eggs were placed into the incubator and kept at 37 °C for 6 days. Then, a window was made in the shell and 30 mL of ASC conditioned medium were added. The windows were sealed with medicinal glue BF-6 (Vertex, Saint-Petersburg, Russia) and the incubation was continued for extra 24 h. The embryos were fixed with 4% paraformaldehyde/2% glutaraldehyde solution in PBS and stained with Carracci’s hematoxylin. Then, the vascularization of CAM was analyzed by morphometric analysis using AngioQuant software (available online: www.cs.tut.fi).

### 4.12. Cytokine and Chemokine Profiling in Conditioned Medium

The concentration of paracrine mediators in conditioned medium (CM) samples was determined using multiplexed fluorescent bead-based immunoassay detection (MILLIPLEX^®^ MAP system, Merck Millipore, Darmstadt, Germany). The panel contained antibody-conjugated beads for the following cytokines and chemokines: EGF, FGF-2, eotaxin, TGF-a, G-CSF, Fit-3L, GM-CSF, fractalkine, IFN-a2, IFNg, GRO, IL-10, MCP-3, IL-12p40, MDC, IL-12p70, PDGF-AA, IL-13, PDGF-AB/BB, IL-15, sCD40L, IL-17A, IL-1RA, IL-1a, IL-9, IL-1b, IL-2, IL-3, IL-4, IL-5, IL-6, IL-7, IL-8, IP-10, MCP-1, MIP-1a, MIP-1b, RANTES, TNFa, TNFb, VEGF. For each assay, a curve was obtained based on different concentrations of cytokine standards, which were examined in the same way as the conditioned medium samples. All samples were measured undiluted. Cytokines and chemokines whose concentrations were reliably detected in the CM samples were selected for analysis.

### 4.13. Magnetic Immunoseparation of EA.hy926 Cells and MSCs

The co-culture for analyzing the transcriptional activity of target genes was pre-trypsinized and separated by magnetic immunoseparation (Miltenyi Biotec, Bergisch Gladbach, Germany). For this purpose, the EA.hy926 cell and MSC suspensions were mixed with magnetic microparticles immobilized with CD31 antibodies to the endothelial marker PECAM (Miltenyi Biotec, Bergisch Gladbach, Germany) and incubated for 15 min at +4 °C. After incubation, the cell suspension was washed to remove unbound particles, resuspended in fresh serum-free culture medium, and placed on a column fixed in a magnet. Microparticles bound to CD31+(EA.hy926 cell) were retained in the magnetic field, resulting in the passage of a suspension of unbound cells (MSCs, CD31-) through the pores of the column. The cells were then collected by centrifugation, the purity of the isolation was determined by flow cytometry, and the remaining suspension was used for further analysis.

### 4.14. Quantitative PCR (qPCR) Analysis of Gene Expression

qPCR analysis was used to assess changes at the transcriptional level. Gene expression analysis was performed using 1 × 10^6^ cells per sample for EA.hy926 cells and 0.5 × 10^6^ cells per sample for MSCs. Total RNA was isolated using ExtractRNA reagent (Eurogen, Moscow, Russia) with homogenization in lysis buffer for 5 min at room temperature. RNA quality and concentration were assessed spectrophotometrically (NanoDrop 2000, Thermo Fisher Scientific, USA), with an A260/A280 ratio of 1.9–2.0. To remove traces of genomic DNA, samples were treated with Ambion DNase I (RNase-free) (Thermo Fisher, USA) according to the manufacturer’s protocol. cDNA synthesis was carried out using 50 ng of total RNA and the MMLV RT kit (Eurogen, Russia) with a mixture of random hexamer and oligo (dT) primers. A no-reverse-transcription control (no-RT control) was included in each experiment to exclude potential genomic DNA contamination. qPCR reactions were performed in a total volume of 20 µL using qPCRmix-HS SYBR (Eurogen, Russia) on an Mx3000P instrument (Stratagene, San Diego, CA, USA). Cycling conditions were as follows: 95 °C for 3 min, followed by 40 cycles (95 °C for 15 s, 60 °C for 30 s, 72 °C for 30 s), with a subsequent melting curve analysis. Each sample was analyzed in at least three technical replicates. Expression of the *GJA1*, *HIF1A*, *VEGF*, *FGF2*, *CXCL12*, *IL6*, *IL8*, and *CCL2* genes was analyzed using the corresponding primers (Qiagen, Hilden, Germany). The *ACTB* and *RPLP0* genes were used as controls, as their transcription remains stable under various conditions, to determine the relative changes in expression levels of the target genes. Normalized gene expression was calculated using the 2^−ΔΔCt^ method [61].

### 4.15. Statistical Analysis

All experiments were performed with four biological replicates and two technical replicates per sample (three technical replicates for qPCR). Except for the western blot analysis, all other experiments were performed using a single biological replicate, which constitutes a limitation of the study. The data are presented as mean ± standard deviation (mean ± SD) to illustrate variability between biological replicates. Statistical analysis was performed using Microsoft Excel 2016 and GraphPad Prism 9 software. The nonparametric Mann–Whitney test was applied for small and medium sample sizes (*n* ≤ 30) at a significance level of *p* ≤ 0.05.

## 5. Conclusions

The results of this study confirm the hypothesis that functional GJs mediated by Cx43 play a key role in coordinating the angiogenic response during the interaction of MSCs and EA.hy926 (an Endothelial Cell Model) under conditions of acute hypoxic stress. It has been shown that hypoxia is accompanied by a significant increase in the expression and nuclear translocation of HIF-1α, enhanced cell migration activity, and increased secretion of proangiogenic factors (VEGF, FGF-2, PDGF-AA, GRO, etc.), which indicates the activation of typical adaptive programs aimed at maintaining cellular homeostasis and stimulating angiogenesis. Blockade of GJs with the specific inhibitor CBX led to a decrease in HIF-1α and GJA1 expression, an increase in the level of phosphorylated Cx43 (Ser368), and a disruption of intercellular signal transmission. This was accompanied by inhibition of proliferation and suppression of undirected cell migration.

Analysis of angiogenic potential showed that, while maintaining functional GJs, hypoxia enhances the secretion of mediators that stimulate migration, tubulogenesis, and vascular network development in the CAM model, whereas their blockade completely eliminates these effects. This indicates that the full realization of the angiogenic response requires the preservation of intercellular coordination through GJs, which ensure the synchrony of signaling and metabolic processes between MSCs and EA.hy926, acting not only as structural elements of intercellular communication, but also as active regulators of the hypoxic response and paracrine interaction, supporting the angiogenic potential and adaptation of cells to acute oxygen deficiency. Promising areas for further research include: elucidating the molecular mechanisms of GJs involvement in the regulation of HIF-mediated pathways, developing strategies for targeted modulation of GJs in ischemic and inflammatory tissue damage, and studying the role of intercellular coordination via GJs in the angiogenic potential of various types of primary endothelial cells and MSCs with the aim of expanding the application of the obtained data in regenerative medicine. The next step should be to conduct studies on primary cultures of endothelial cells and MSCs to confirm the results obtained and evaluate intercellular coordination in a more physiologically relevant model.

## Figures and Tables

**Table 1:** Assessment of viability and cell death pathways in MSCs and EA.Hy926 cells after co-culture under hypoxic stress and gap junction blockade.

Viability and Cell Death Pathways	EA.hy926 Cells			Mesenchymal Stromal Cells		
	Control	0.1%	0.1% + CBX	Control	0.1%	0.1% + CBX
Viable (Ann−/PI−),%	91.1 ± 3.2	85.2 ± 3.7	79.9 ± 2.9	92.8 ± 2.8	89.3 ± 2.6	87.8 ± 1.9
Apoptosis (Ann+/PI−),%	4.2 ± 1.1	5.5 ± 2.1	6.4 ± 11.8	3.3 ± 1.1	6.5 ± 1.1	4.9 ± 1.3
Necrosis (Ann−/PI+),%	1.7 ± 0.7	3.9 ± 1.3	8.1 ± 2.5	1.9 ± 0.4	1.1 ± 0.5	2.1 ± 0.3
Post-apoptotic necrosis (Ann+/PI+),%	3.1 ± 1.2	5.4 ± 1.9	5.6 ± 1.1	2 ± 0.6	3.1 ± 0.6	5.2 ± 1.5

## Data Availability

The original contributions presented in this study are included in the article. Further inquiries can be directed to the corresponding author.

## Supplementary Materials

The following supporting information can be downloaded at: https://www.mdpi.com/article/10.3390/ijms262211239/s1.
