# Supplementary material for: The Role of Gap Junctions in MSC-EA.hy926 (An Endothelial Cell Model) Crosstalk Under Hypoxic Stress: Regulation of the Angiogenic Response
Source: Int J Mol Sci. 2025 Nov 20;26(22):11239. doi: 10.3390/ijms262211239 (PMC12653098; doi:10.3390/ijms262211239)
Supplement: Supplementary file 1 [file ijms-26-11239-s001.zip › ijms-3959274-supplementary.pdf]

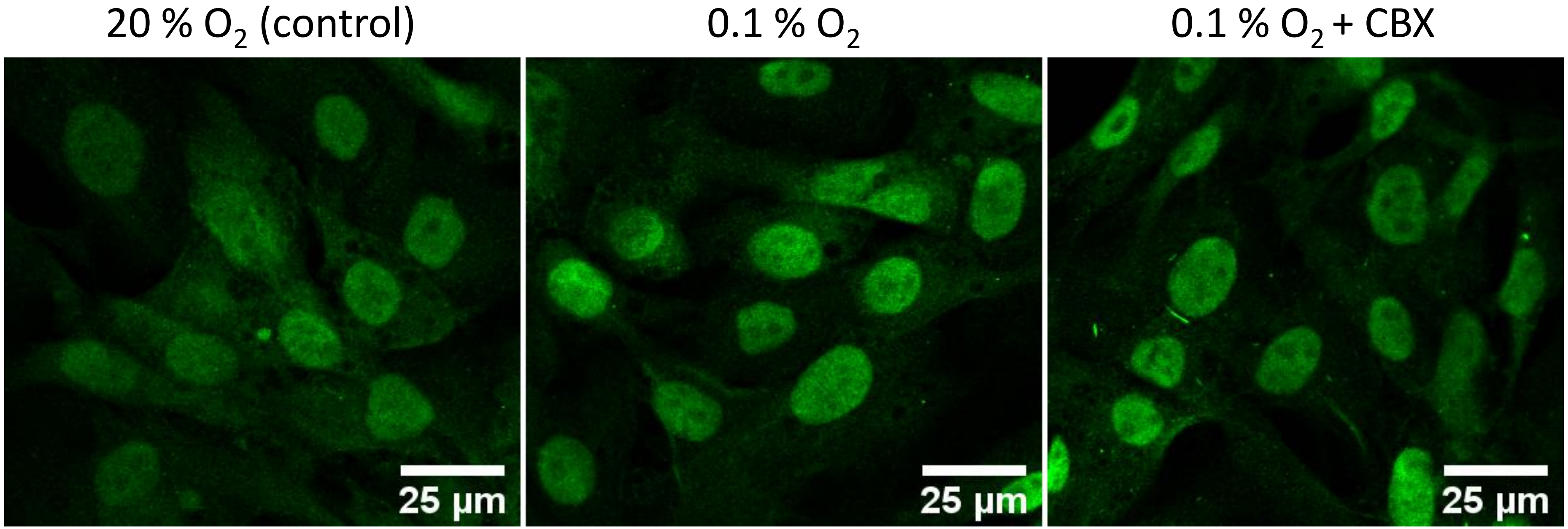

Figure S1. Immunofluorescent staining of HIF-1α in co-culture cells (MSCs + EA.Hy926) analyzed by confocal microscopy. Representative microphotographs. CBX - carbenoxolone, a specific gap junction inhibitor.

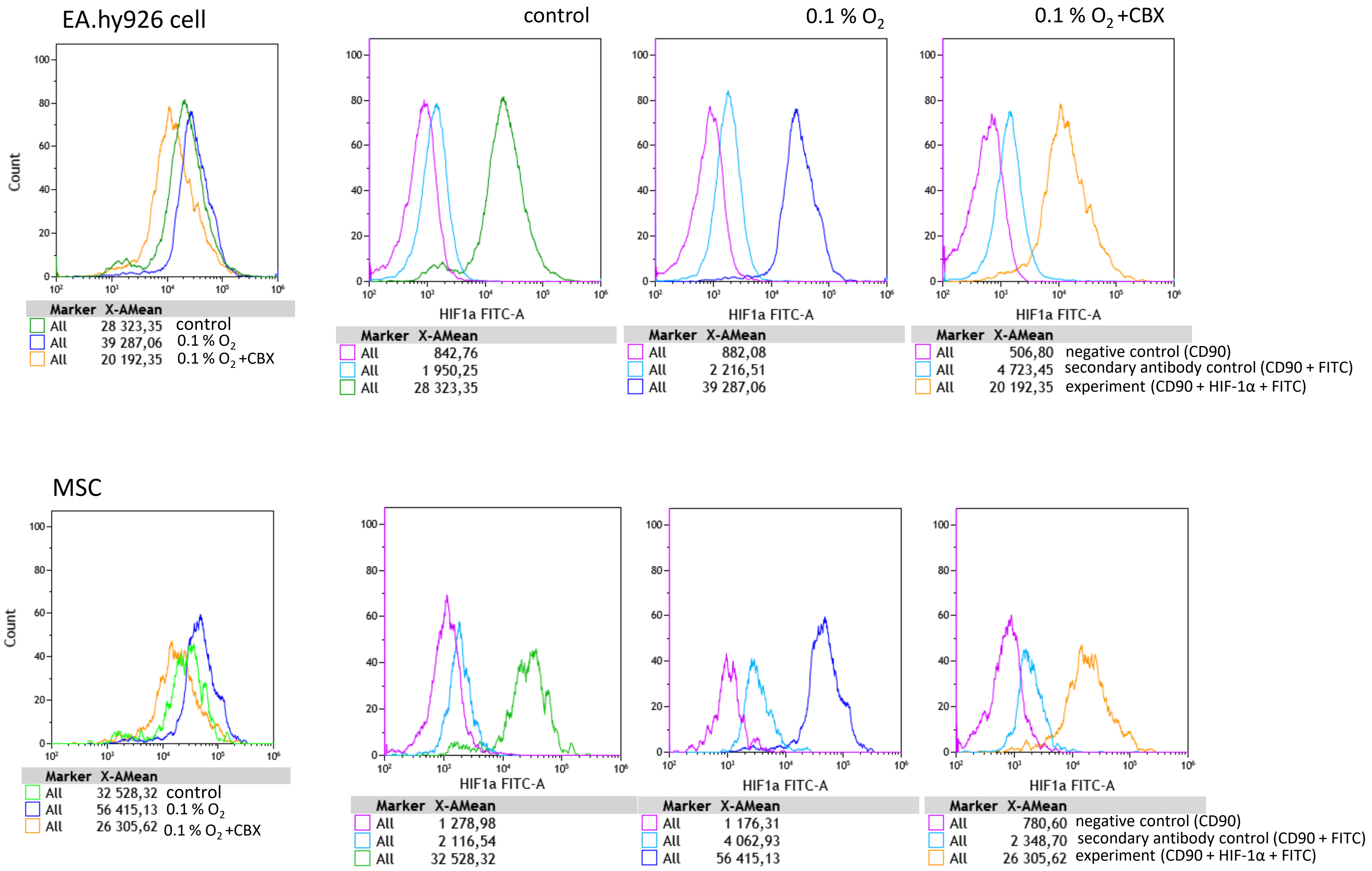

Figure S2. Representative histograms, flow cytometry. Distribution of cells based on mean fluorescence intensity (MFI). HIF-1α protein level in hypoxia-treated cells stained with anti-HIF-1α antibodies. 1. negative control, 2. secondary antibody control, 3. experiment. CBX - carbenoxolone, a specific gap junction inhibitor.

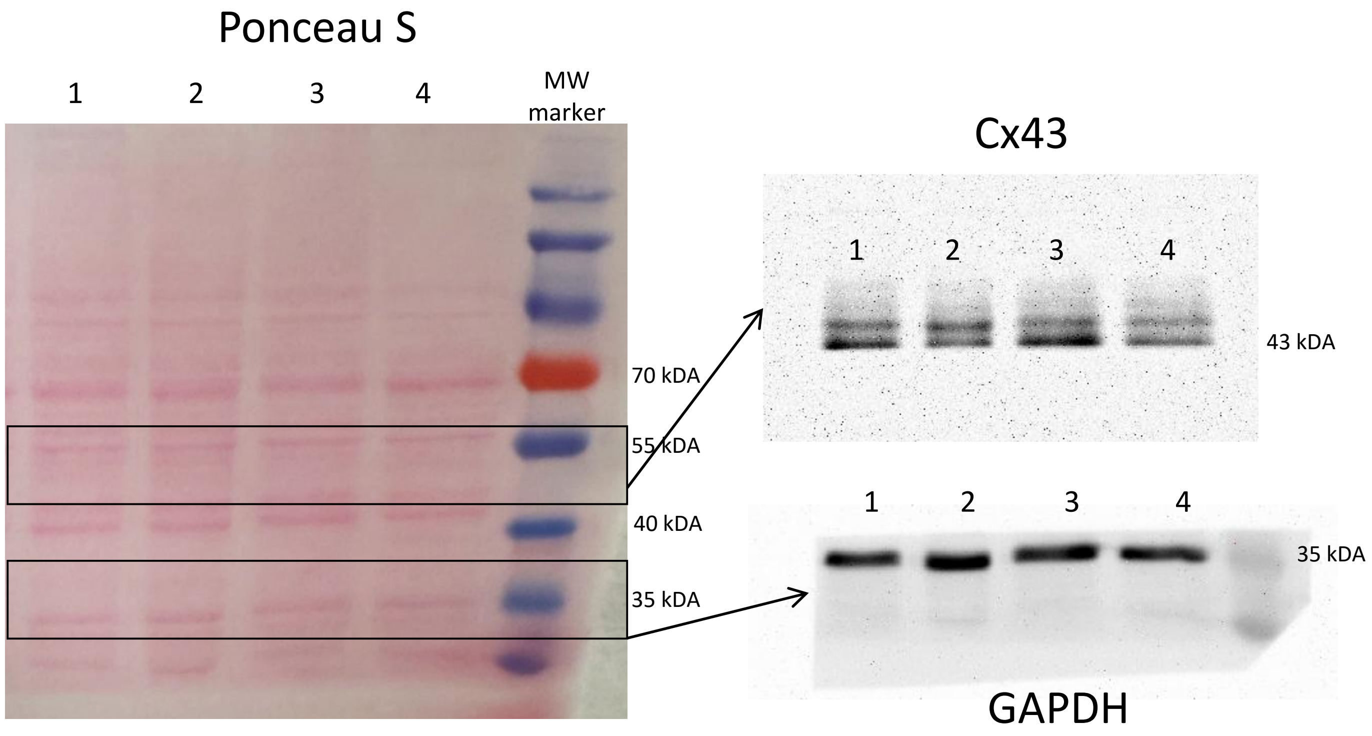

Figure S3. Representative Western Blot analysis of Cx43 expression in co culture cells. (a) - Ponceau S staining; (b) -Immunoblots for Cx43 and GAPDH proteins. 1 - control; 2 - data for 20% O_2 + CBX are not presented in the article; 3 – 0.1 % O_2; 4 – 0.1 % O_2 + CBX. CBX-carbenoxolone, a specific gap junction inhibitor.

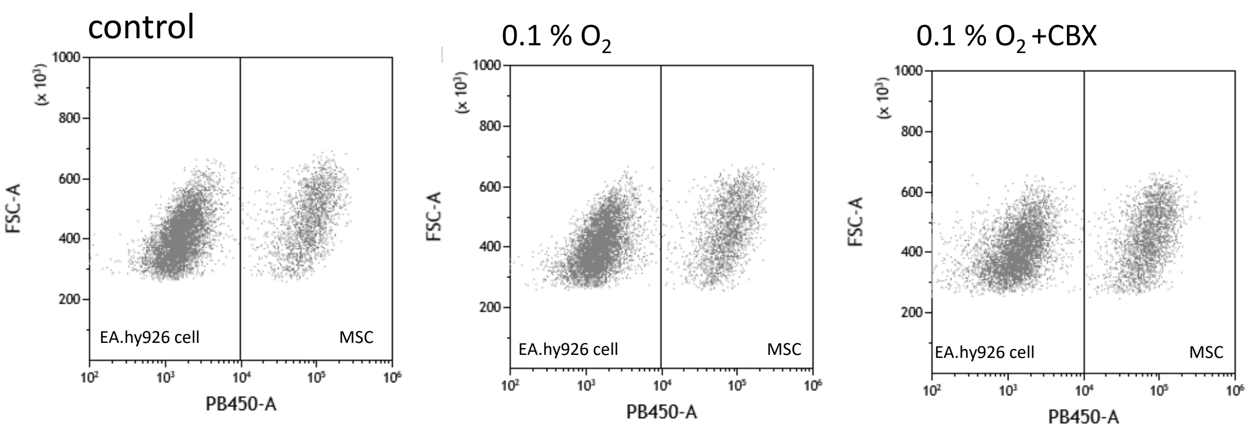

Figure S4. Cell viability analysis of MSC and EA.hy926 in co-culture. For identification of MSCs, anti-CD90-PB450 (Beckman Coulter Life Sciences, USA) antibody was used. Unstained cells correspond to the EA.hy926 cell population. Representative dot-plot. CBX - carbenoxolone, a specific gap junction inhibitor.

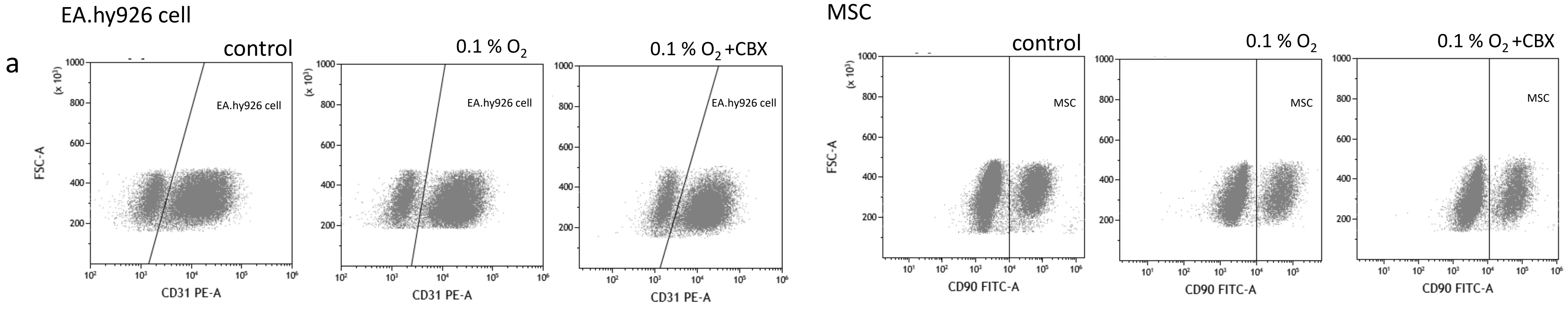

Figure S5. Cell Cycle Analysis of MSC and EA.hy926 in co-culture. a - Cells were first stained with antibodies against surface markers to identify cell types: MSC — CD90-FITC (Sony, Japan), EA.hy926 — CD31-PE (Beckman Coulter Life Sciences, USA). Each antibody was applied to a separate sample. Flow cytometry allowed determination of cell cycle phase distribution for each population while maintaining correct identification of MSCs and EA.hy926 cells. Representative dot-plot.
